# End fire linear antenna array synthesis using differential evolution inspired Adaptive Naked Mole Rat Algorithm

**DOI:** 10.1038/s41598-023-39509-4

**Published:** 2023-07-29

**Authors:** Harbinder Singh, Nitin Mittal, Amit Gupta, Pratap Singh, Fikreselam Gared

**Affiliations:** 1 Department of Electronics and Communication Engineering, UCRD, Chandigarh University, Punjab, India; 2 Skill Faculty of Engineering and Technology, Shri Vishwakarma Skill University, Dudhola, Haryana India; 3 Department of Electronics and Communication Engineering, I.K Gujral Punjab Technical University, Punjab, India; 4 Department of Computer Science and Engineering, Guru Jambheshwar University of Science and Technology, Hisar, Haryana India; 5 Faculty of Electrical and Computer Engineering, Bahir Dar Institute of Technology, Bahir Dar University, Bahir Dar, Ethiopia

**Keywords:** Engineering, Mathematics and computing

## Abstract

Linear antenna arrays (LAAs) play a critical role in smart system communication applications such as the Internet of Things (IoT), mobile communication and beamforming. However, minimizing secondary lobes while maintaining a low beamwidth remains challenging. This study presents an enhanced synthesis methodology for LAAs using the Adaptive Naked Mole Rat Algorithm (ANMRA). ANMRA, inspired by mole-rat mating habits, improves exploration and exploitation capabilities for directive LAA applications. The performance of ANMRA is assessed using the CEC 2019 benchmark test functions, a widely adopted standard for statistical evaluation in optimization algorithms. The proposed methodology results are also benchmarked against state-of-the-art algorithms, including the Salp Swarm Algorithm (SSA), Cuckoo Search (CS), Artificial Hummingbird Algorithm (AHOA), Chimp Optimization Algorithm (ChOA), and Naked Mole Rat Algorithm (NMRA). The results demonstrate that ANMRA achieves superior performance among the benchmarked algorithms by successfully minimizing secondary lobes and obtaining a narrow beamwidth. The ANMRA controlled design achieves the lowest Side Lobe Level (SLL) of − 37.08 dB and the smallest beamwidth of 74.68°. The statistical assessment using the benchmark test functions further confirms the effectiveness of ANMRA. By optimizing antenna element magnitude and placement control, ANMRA enables precise primary lobe placement, grating lobe elimination, and high directivity in LAAs. This research contributes to advancing smart system communication technologies, particularly in the context of IoT and beamforming applications, by providing an enhanced synthesis methodology for LAAs that offers improved performance in terms of secondary lobe reduction and beamwidth optimization.

## Introduction

Long-range, high-efficiency wireless communication systems hold significant promise for a wide range of Internet of Things (IoT) applications in smart cities. Advanced wireless communication systems, such as those incorporating massive multi-input–multi-output (MIMO) and smart antenna system tracking technologies, have emerged as promising solutions to enhance system capacity and spectral efficiency^1^. One key aspect of improving wireless system performance is enhancing the beam directivity of antennas, which can be achieved through the use of directional antennas and antenna arrays^2^. However, relying solely on directional antennas in wireless communication systems can introduce drawbacks, such as the presence of back lobes despite having low Side Lobe Levels (SLL)^3^. Additionally, real-time applications often require beam control performance, such as beamforming, which involves adjusting various parameters of the antenna array, including feeding element magnitude, position, spacing, and phase^4^. These factors do not exhibit a simple linear relationship, making the optimization of the antenna array's beam pattern a critical challenge for researchers. Furthermore, there exists a trade-off between sidelobe and primary lobe beamwidth in the radiation pattern of an antenna array^5^. This trade-off implies that reducing sidelobes alone may result in a wider primary lobe beamwidth, leading to reduced energy transmission efficiency. Thus, developing an efficient and effective method for synthesizing the radiation pattern of an antenna array is of utmost importance.

An antenna array is a collection of radiating antenna elements that are placed and phased in such a way as to control the radiation pattern for desired characteristics. The geometrical and electrical arrangement of the antenna array is arranged in such a way to generate constructive interference in the intended direction while producing destructive interference in the unwanted direction. As a result, in addition to providing high directivity, they can also control the primary lobe and nulls. Such characteristics are critical in IoT^6^, cognitive radio^7^, phased array^8^, fifth-generation (5G) communication^9^ and many other applications. An antenna array can be configured in a variety of ways, linear antenna array (LAA) is a form of an antenna array that is employed in many real time applications because of its easy construction and feeding mechanism. Antenna array synthesis is a nonlinear and complicated problem to solve. There are many traditional ways of reducing SLLs in antenna arrays, including the Chebyshev^10^, Binomial^11^, and Taylor^12^ approaches. Gradient techniques require an initial assumption in order to achieve a decent solution and can become trapped in local minima^13^. Meta-heuristic algorithms, on the other hand, have grown in popularity for providing speedy, low-cost, and reliable solutions to complex optimization problems. When compared to a typical deterministic technique, they do not require any gradient data and are straightforward and easy to implement^14^.

Nature-inspired algorithms can be broadly classified into two categories: Evolutionary Algorithms (EA) and Swarm Intelligent (SI) algorithms. EAs are based on Darwin's theory of natural selection and mimic the process of species evolution. On the other hand, SI algorithms draw inspiration from the collective behavior of organisms such as ants, birds, bees, monkeys, and wolves, who work together towards a common goal. SI algorithms leverage the learning ability and addictiveness of these organisms to address complex real-world problems. With the diverse range of applications for nature-inspired algorithms, researchers have devoted their efforts to enhancing their efficacy and adaptability in addressing various optimization challenges. For example, in the study conducted by^15^, they developed a binary technique based on Differential Evolution (DE) that integrated innovative components such as unique solution representations, mapping techniques and diversity procedures. Similarly,^16^ introduced a Genetic Algorithms (GA)-based technique with multiple modifications specifically tailored to handle high-dimensional and computationally expensive problems. A distributed individuals DE technique that operates on a distributed person’s architecture is addressed in^17^, specifically designed for scenarios involving multiple peaks. In the realm of feature selection, Song et al.^18^ presented a variable-size cooperative co-evolutionary Particle Swarm Optimization (PSO) method. Furthermore, Peng et al.^19^ introduced a courtship learning framework to boost the performance of the Firefly Algorithm (FA).

As researchers strive to efficiently address real-world complex problems, they actively explore new optimization approaches that can yield satisfactory results and overcome the challenges associated with getting stuck in local minima. Antennas are essential components for several IoT applications. Several types of antenna array, including those used for 5G, Sub-6G, beamforming and point-to-point communications, find widespread usage in smart city applications. In this paper, an effective optimization algorithm named Adaptive Naked Mole Rat Algorithm (ANMRA) is suggested for the optimization issue of LAA. The NMRA mimics the naked mole rats (NMRs) behavioral characteristics^20^. ANMRA is based on the DE variant (DE/rand/2)^21^ and the attraction–repulsion strategy to solve the problems of poor optimization accuracy and the tendency to fall into a local optimum in NMRA. When trying to find the best forward path in the original NMRA, it's easy to get stuck in a local optimum. With the DE/rand/2 variant, the algorithm's exploration and optimization abilities can be effectively increased, as well as its ability to jump out of a local optimum. As a result of the combination of the global best NMR individual attraction and the global worst NMR individual repulsion, the algorithm's population is more diverse and optimized, and the search solution space is larger when the attraction–repulsion strategy algorithm is employed in the exploitation stage.

The following are the key accomplishments of this research:

- The LAA optimization problem is designed for primary lobe placement, grating lobe elimination, low SLL, narrow beamwidth, and high directivity.
- To develop speedy, low-cost, and robust solutions, an adaptive optimization technique is proposed.
- The real life applicability of the proposed algorithm is validated on LAA for antenna element magnitude or/and placement control. The proposed ANMRA performance is also assessed using the CEC 2019 benchmark test functions.
- The findings suggest that feeding magnitude can be used to effectively minimize SLL while position control can be used to manage beamwidth. Controlling amplitude and location at the same time results in a narrow beamwidth along with negligible SLL.
- The 3D radiation pattern findings are also validated in order to authenticate the presence of subsidiary and grating lobes.

The paper is organized into five sections to provide a coherent structure for the research. “Introduction” section establishes the theoretical background and conducts a comprehensive literature review to contextualize the study. “Problem formulation for end fire LAA” section focuses on mathematically framing the LAA problem, outlining the objectives and challenges involved. “ANMRA algorithm” section involves an extensive modeling analysis of the ANMRA algorithm which provided detailed descriptions of the methodology and the benchmark tests conducted. This modeling provides a deep understanding of the algorithm's behavior and capabilities. “Results analysis” section conducts a detailed assessment of the ANMRA results, comparing them to other approaches to assess strengths and weaknesses. Key findings and their effects are summarized in “Conclusion” section to conclude the study.

## Problem formulation for end fire LAA

**Figure 1:**
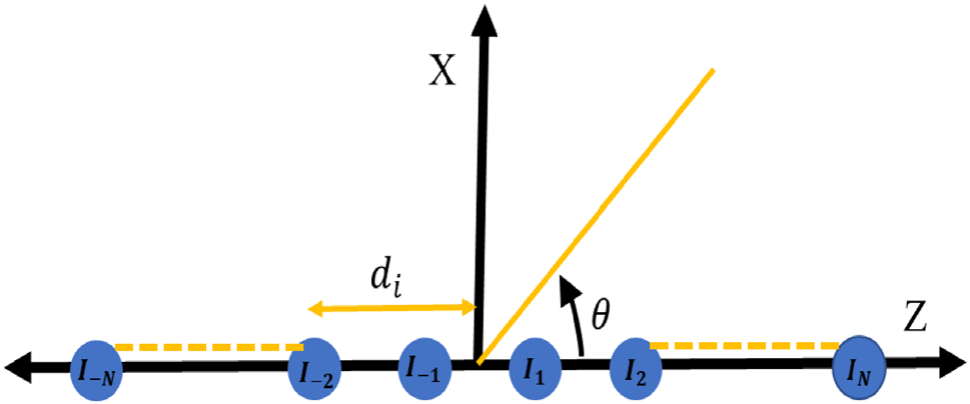
Structure of LAA.

An LAA is a specific configuration of array design where radiating elements are arranged in a linear sequence. This arrangement typically involves an even number of radiating antennas *(2N)* aligned in a linear sequence along the Z-axis^22^, as depicted in Fig. 1. The Array Factor (*AF*) for such arrangements can be mathematically formulated as follows:

1 $$AF\left(LAA\right)=\sum_{\begin{array}{c}n=-N\\ N\ne 0\end{array}}^{N}{E}_{n}{e}^{j\left[\beta {z}_{n}\mathrm{cos}\left(\theta \right)+{\alpha }_{n}\right]}$$

where ${E}_{n}$, ${\alpha }_{n}$ and ${z}_{n}$ are the feeding magnitude, phase and location of the radiating antenna elements and $\beta$ is the wavenumber. It is further assumed that the geometry of the structure is symmetric along the array axis (Z-axis) i.e. ${E}_{n}={E}_{-n}$ and ${\alpha }_{n}={\alpha }_{-n}$ then the *AF* can be simplified as

2 $$AF\left({I}_{n},{z}_{n},{\alpha }_{n}\right)=2\sum_{n=1}^{N}{E}_{n}\mathrm{cos}[\beta {z}_{n}\mathrm{cos}\left(\theta \right)+{\alpha }_{n}]$$

where ${\alpha }_{n}=-\beta {z}_{n}\mathrm{cos}\left({\theta }_{0}\right)$ maybe used to adjust maximum radiation direction. The proposed approach is tested on the End Fire Array (EFA) pattern of LAA, which features a major lobe parallel to the array axis ${(\theta }_{0}={0}^{0} or {\theta }_{0}={180}^{0})$. This design is analyzed for EFA radiation towards zero degrees elevation. Thus, the *AF* may be summarized in terms of EFA pattern with the main lobe towards zero degrees elevation as:

3 $$AF\left({I}_{n},{z}_{n}\right)=2\sum_{n=1}^{N}{E}_{n}\mathrm{cos}\left\{\beta {z}_{n}[\mathrm{cos}\left(\theta \right)-1]\right\}$$

The primary goal of the research is to reduce SLL while increasing directivity. This can be accomplished by predicting the array elements' regulated magnitude or/and locations for an objective function $(OF)$ of

4 $$OF=\frac{\left|AF\left({E}_{n},{z}_{n},\theta \right)\right|}{\left|AF\left({E}_{n},{z}_{n}, {\theta }_{0}\right)\right|}+\frac{1}{DIR({E}_{n},{z}_{n}, {\theta }_{0})}$$

The initial part of the $OF$ is related to the SLL, while the second part focuses on achieving high directivity. In wireless communications, the goal is not only to minimize the SLL but also to achieve optimal directivity. Improving directivity results in a narrower first null beam width (FNBW). Therefore, reducing the combined $OF$ leads to favorable conditions of low SLL, narrow FNBW, and strong directivity.

## ANMRA algorithm

### A. Naked Mole Rat Algorithm

The NMRA mimics the NMRs' behavioral characteristics^20^. NMRs matting patterns can be accurately simulated using NMRA. NMRs live in groups of 70–80 entities, with the queen as the leader and the rest of the group as workers and breeders.

Productive NMRs, whose primary job is to mate with the queen, are known as breeders, while the remaining NMRs perform a wide range of other duties of the colony. There are three steps in the NMRA process. First, a group of NMRs is created and then separated into workers and breeders pool to execute the worker and breeder phase. The details of each step are discussed as follows:

#### Initialization

A population of *n* NMRs is generated randomly with each NMR represented as a D-dimensional vector in a vector space. Each NMR must be initialized as follows:

5 $${NMR}_{i,j}= {NMR}_{min,j}+U\left(\mathrm{0,1}\right)\times \left({NMR}_{min,j}- {NMR}_{max,j}\right)$$

here $i\in [\mathrm{1,2},\dots \dots ,n]$, $j\in [\mathrm{1,2},\dots \dots ,D]$, ${NMR}_{i,j}$ is the ${i}{th}$ solution in the ${j}{th}$ dimension, ${NMR}_{min,j}$ and ${NMR}_{max,j}$ are the lower and upper limit of the problem under consideration, and $U\left(\mathrm{0,1}\right)$ is a random number generated between 0 and 1. The performance of each solution is assessed on the basis of the fitness value attained after initialization. On the basis of fitness value attained, the best NMR ${(d}_{best})$ is determined and the rest NMRs are classified into breeders (B) and workers (W) pool.

#### Worker phase

During this phase, in order to pursue as a breeder, the workers (W) try to improve their fitness. The search equation used to create the updated solution is as follows.

6 $${w}_{i}^{t+1}= {w}_{i}^{t}+{ \lambda }_{m}({w}_{j}^{t}-{w}_{k}^{t})$$

where ${w}_{i}^{t}$ represents ${i}{th}$ worker in ${t}{th}$ iteration, ${w}_{i}^{t+1}$ represents updated fitness solutions and ${w}_{j}^{t},{w}_{k}^{t}$ are the randomly chosen NMRs from the workers' pool. The ${\lambda }_{m}$ is the random variable uniformly distributed $[\mathrm{0,1}]$.

#### Breeder phase

Breeder (B) needs to enhance its fitness before it can be considered a mating partner or even retain its breeder status. The breeders are updated on the basis of breeding probability (${b}_{p}$). The breeder may be restored into the worker pool once the breeder is unable to outperform its fitness.

7 $${b}_{i}^{t+1}=\left(1-{ \lambda }_{m}\right){b}_{i}^{t}+{ \lambda }_{m}\left({d}_{best}-{b}_{i}^{t}\right)$$

where ${b}_{i}^{t}$ signifies ${i}{th}$ breeder in ${t}{th}$ iteration, and the mating frequency is controlled by the parameter ${\lambda }_{m}$ (initially set to 0.5).

Until the termination criteria are met, this process is repeated iteratively. If you're looking for the best breeder in the entire population, that's a possibility. The optimum solution to the problem under consideration is the breeder with the best fitness value.

NMRA has recently attracted the interest of researchers for its simplicity and linearity. To achieve greater efficiency and better performance, adaptive NMRA (ANMRA) seeks to improve both the fundamental exploitation and exploration capabilities of the basic NMRA.

### B. Adaptive Naked Mole Rat Algorithm

#### Worker phase

In the worker phase, the NMRs population is divided into two sections. The final solution is evaluated using two different search strategies in order to improve the exploration efficiency of NMRA. For the first half of the population, we use the search equation similar to the standard NMRA search equation:

8 $${w}_{i}^{t+1}={w}_{i}^{t}+{ \lambda }_{m}\left({w}_{j}^{t}-{w}_{k}^{t}\right)$$

For the 2nd part of the population, the DE inspired search equation (DE/rand/2) is used and is given by

9 $${w}_{i}^{t+1}={w}_{i}^{t}+{ \lambda }_{m}\left(\left({w}_{j}^{t}-{w}_{k}^{t}\right)+({w}_{p}^{t}-{w}_{q}^{t})\right)$$

where ${w}_{j}^{t},{w}_{k}^{t},{w}_{p}^{t},{w}_{q}^{t}$ are the randomly chosen NMRs from the workers' pool.

#### Breeder phase

New breeders' search directions are updated by the best NMR estimated in the basic NMRA breeder phase. Premature convergence occurs when the globally optimal individuals collapse into local optima. As a result, it is necessary to make use of the guiding function of the better individuals closer to the optimal solution and/or farther from the worst solution in order to reduce the likelihood of the algorithm reaching the local optimal. In this work, an attraction–repulsion strategy has been employed to address the issue at hand.

In the modified breeder phase, the attraction–repulsion strategy is utilized using both global best and global worst solutions, allowing the breeder population to move randomly under the influence of both attraction and repulsion to arrive at a solution that is optimal. The premature phenomenon will occur as the iterative process of the algorithm enters the later stages. At this point, the introduction of the global worst position can help to improve the diversity of the population. It broadens the scope of local search and eliminates the premature convergence problem. Using the attraction–repulsion strategy based search equation, breeders can find their ideal position as follows:

10 $${b}_{i}^{t+1}=\left(1-{ \lambda }_{m}\right){b}_{i}^{t}+{ \lambda }_{m}[{\alpha }_{1}\left({d}_{best}-{b}_{i}^{t}\right)-{\alpha }_{2}\left({d}_{worst}-{b}_{i}^{t}\right)]$$

where ${d}_{best}$ and ${d}_{worst}$ are the optimal or best position and worst position of NMRs respectively.

#### Parametric adaptations

Here, to make the algorithm self-adaptive, three essential parameters namely mating factor $({ \lambda }_{m}$), ${\alpha }_{1}$ and ${\alpha }_{2}$ are made adaptable so that no user-based tunning is required for these parameters.

Firstly, parameter ${\lambda }_{m}$ is incorporated with simulated annealing (SA) mutation operator^23^ and defined as:

11 $${\lambda }_{m}={\uplambda }_{min}+\left({\uplambda }_{max}-{\uplambda }_{min}\right)\times {d}^{\left(s-1\right)}$$

where $d$ is set to 0.95, ${\lambda }_{max}$, ${\uplambda }_{min}$ and s are randomly generated values in the range [0,1].

Secondly, parameter ${\alpha }_{1}$ associated with the global best solution and make it adaptive using the natural exponent mutation operator^24^. The mathematical equation to model ${\alpha }_{1}$ is defined as:

12 $${\alpha }_{1}={\alpha }_{min}+\left({\alpha }_{max}-{\alpha }_{min}\right){e}^{-[\frac{t}{\frac{{t}_{max}}{10}}]}$$

where ${\alpha }_{min}$ is set to 0.5, the value of ${\alpha }_{max}$ is taken as 0.9, $t$ and ${t}_{max}$ are defined as the current iteration and maximum iterations value respectively. This mutation operator helps the algorithm to converge faster than linear strategies in the early stages of the search process and produce superior results for most problems involving continuous optimization.

Finally, an important parameter ${\alpha }_{2}$ incorporated with the worst solution (${d}_{worst}$) and follows linearly decreasing distribution^25^ ranging from 0.5 to 0.1. The parameter ${\alpha }_{2}$ is defined as:

13 $${\alpha }_{2}= {\alpha }_{max}-(\frac{{\alpha }_{max}- {\alpha }_{min}}{{t}_{max}})\times k$$

where value of ${\alpha }_{max}$ is taken as 0.5, ${\alpha }_{min}$ is set to 0.1 and $k$ is randomly distributed in the range [0, 1]. The pseudo-code of ANMRA is shown in Algorithm 1.

**Figure:**
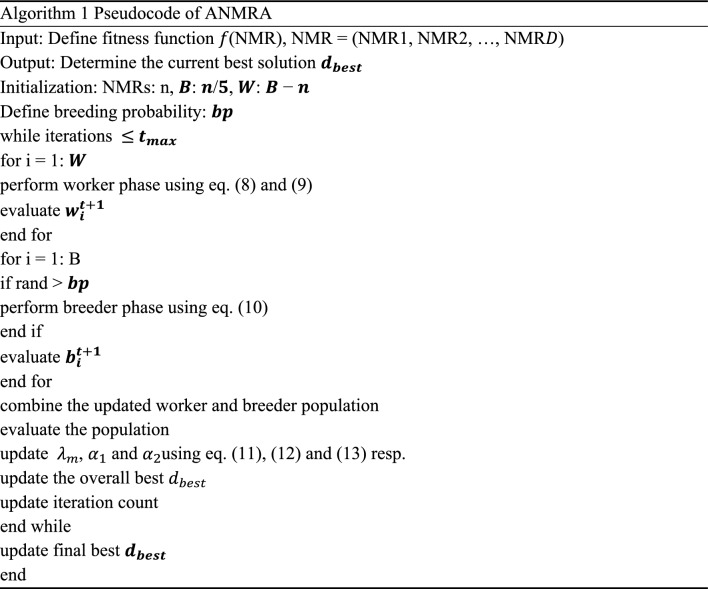

## Results analysis

### Statistical testing For CEC2019 test suite

**Figure 2:**
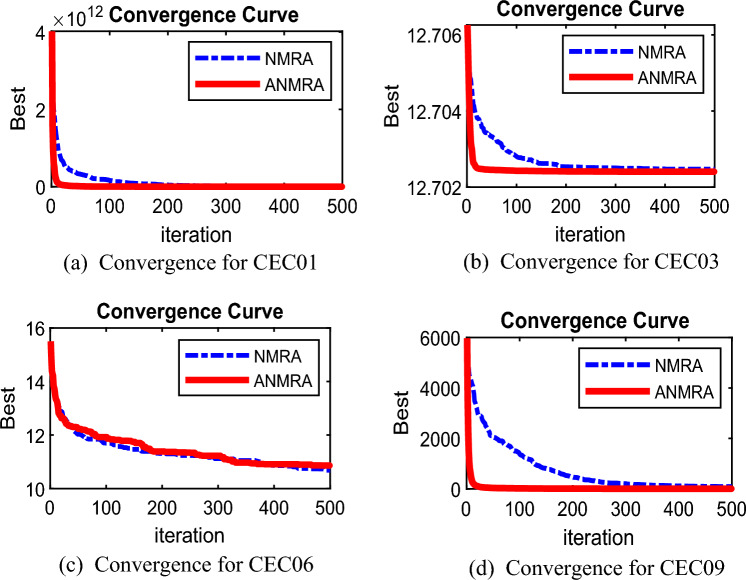
Convergence graph for CEC2019 benchmark functions.

The "100-Digit Challenge"^26^a collection of ten CEC 2019 benchmark test functions, has been opted for ANMRA assessment. The effectiveness of ANMRA is evaluated by comparing it to jDE100^26^, DE^21^, Fitness-Dependent Optimizer (FDO)^27^, Self-Adaptive Salp Swarm Algorithm (ASSA)^28^ and NMRA. MATLAB version R2020a was used to simulate the ANMRA and competitive algorithms. With the support of 30 agents, each algorithm goes through 500 iterations. For each of the methods under discussion, the results are presented in terms of the mean and standard deviation values for 30 independent runs. ANMRA outperforms rival algorithms in all except CEC02, CEC(05-07), and CEC(9-10) test functions (Table 1). Figure 2 shows the NMRA and ANMRA convergence graphs for various test functions.

**Table 1:** Statistical results for CEC 2019 Test Suite.

Function	Metric	jDE100	DE	FDO	ASSA	NMRA	ANMRA
CEC01	Mean	1.59E+05	3.42E+11	1.34E+09	5.57E+05	6.65E+09	**7.51E+04**
	Std	1.60E+05	3.39E+11	1.72E+09	4.12E+05	9.21E+09	**9.45E+04**
	p-rank	−	−	−	−	−	
	f-rank	2	6	4	3	5	1
CEC02	Mean	2.39E+06	4.83E+01	1.73E+01	1.79E+01	1.75E+01	1.73E+01
	Std	2.72E+04	9.29E+01	**1.65E−05**	4.10E−01	7.24E−02	6.24E−05
	p-rank	−	−	+	−	−	
	f-rank	6	5	1	4	3	2
CEC03	Mean	1.31E+06	1.27E+01	1.27E+01	1.27E+01	1.27E+01	1.27E+01
	Std	8.52E+05	1.60E−03	1.40E−11	3.14E−05	4.73E−05	**1.53E−16**
	p-rank	−	−	−	−	−	
	f-rank	6	5	2	3	4	1
CEC04	Mean	3.48E+05	1.55E+03	3.75E+01	2.63E+03	1.15E+03	3.75E+01
	Std	1.15E+05	1.63E+03	2.05E+01	1.06E+03	4.12E+02	**1.92E+01**
	p-rank	−	−	−	−	−	
	f-rank	6	4	2	5	3	1
CEC05	Mean	1.67E+05	2.16E+00	**1.19E+00**	2.49E+00	2.17E+00	1.25E+00
	Std	8.43E+04	3.06E−01	**1.23E−01**	2.91E−01	8.31E−02	2.00E−01
	p-rank	−	−	+	−	−	
	f-rank	3	4	1	6	5	2
CEC06	Mean	3.84E+04	**9.77E+00**	1.10E+01	1.14E+01	1.08E+01	1.09E+01
	Std	2.06E+03	1.49E+00	7.62E−01	9.15E−01	5.72E−01	6.16E−01
	p-rank	−	+	−	−	+	
	f-rank	6	1	4	5	2	3
CEC07	Mean	9.11E+06	1.26E+03	**5.05E+00**	9.08E+02	5.85E+00	5.11E+02
	Std	4.53E+06	4.12E+02	6.27E−01	2.35E+02	5.42E−01	2.78E+02
	p-rank	−	−	+	−	+	
	f-rank	6	5	1	4	2	3
CEC08	Mean	1.22E+09	7.01E+00	4.85E+00	6.15E+00	5.93E+00	**4.83E+00**
	Std	4.39E+08	2.15E−01	7.80E−01	5.84E−01	5.91E−01	**1.83E−01**
	p-rank	−	−	−	−	−	
	f-rank	6	5	2	4	3	1
CEC09	Mean	9.21E+08	2.71E+02	**2.55E+00**	3.69E+02	9.12E+01	4.19E+00
	Std	1.13E+08	3.52E+02	1.54E−01	1.41E+02	6.96E+01	6.57E−01
	p-rank	−	−	+	−	−	
	f-rank	6	4	1	5	3	2
CEC10	Mean	1.54E+06	2.05E+01	**1.93E+01**	2.05E+01	2.04E+01	2.00E+01
	Std	7.46E+05	1.12E−01	3.65E+00	**7.44E−02**	1.55E−01	2.57E+00
	p-rank	−	−	+	−	−	
	f-rank	6	4	1	5	3	2
Average f-rank		5.3	4.3	1.9	4.4	3.3	**1.8**
Overall f-rank		6	4	2	5	3	**1**

Significant values are in bold.

Because of the stochastic nature of the aforesaid algorithms, the performance of the competing algorithms has been statistically tested using the Wilcoxon rank-sum test and the Friedman rank (f-rank) test. The Wilcoxon rank-sum test has been used to get the p-rank when the results of the two methods need to be compared. Here, the proposed ANMRA's statistical efficiency needs to be checked in comparison to other approaches. The rank of different methods for each CEC function is displayed in the fourth row of Table 1 and is expressed in terms of win(w)/loss(l)/tie(t). When the tested approach outperformed the suggested one and is assigned the + sign, it is called the win(w) condition. The algorithm's performance in the loss(l) condition is poorer than in the proposed strategy, hence the − sign is used in this case. In the last tie(t) condition, both procedures under test are statistically significant, as indicated by the = symbol. Table 1 shows that the proposed ANMRA beat competitive algorithms in the majority of cases.

Following the Wilcoxon rank-sum test, the performance of the proposed ANMRA algorithm was further evaluated using the f-rank test. This test assigned a distinct rank to each algorithm based on its performance, and the overall f-rank was calculated for the entire test suite (Table 1). Among all the algorithms analyzed, ANMRA consistently achieved the highest performance and ranked first in most cases.

### B. Analysis of ANMRA for end fire LAA

In this part, the LAA situation is explored utilizing the ANMRA technique for low SLL, small FNBW and high directivity. The LAA can be synthesized with the magnitude or/and location control and the study will assess all potential combinations of regulating LAA. The ANMRA is used to discover the optimal regulating parameters for the LAA issue once it has been set up. The parametric adaptations in ANMRA emphasize exploration in the early phases of iterations, whereas it emphasizes exploitation in the latter stages. The results are validated using the MATLAB analytical software. Iterations are restricted to 500 due to the time constraints imposed by real-time applications and 25 search agents are employed. To eliminate random distortions, the simulations are run 20 times independently and the median results are reported. The findings are compared to those of other comparable approaches such as SSA (Salp Swarm Algorithm), CS (Cuckoo Search), AHOA (Artificial hummingbird algorithm), ChOA (Chimp Optimization Algorithm) and NMRA. The parameters used in various meta-heuristics are shown in Table 2.

**Table 2:** Parameter settings for different algorithm.

Algorithm	Specifications
SSA^29^	NP = 25; N = 10; ${t}_{max}$ = 500; c1 = [2 to 0]
CS^30^	NP = 25; N = 10; ${t}_{max}$ = 500; pa = 0.25
AHOA^31^	NP = 25; N = 10; ${t}_{max}$ = 500; migration coefficient = 2* NP
ChOA^32^	NP = 25; N = 10; ${t}_{max}$ = 500; f = [2.5 to 0]; a = [− 1, 1]; c = [2 to 0]
NMRA^20^	NP = 25; N = 10; ${t}_{max}$ = 500; ${b}_{p}$=0.5, ${\lambda }_{m}$=0.5
ANMRA	NP = 25; N = 10; ${t}_{max}$ = 500; ${b}_{p}$=0.5, ${\lambda }_{m}$= ${\alpha }_{1}$ = ${\alpha }_{2}$= adaptive

Here, NP is the population size, N is the Dimension of the problem or antenna size, ${t}_{max}$ is the maximum number of iterations.

#### 1) LAA for magnitude control only

The result analysis begins with the examination of magnitude only control of feeding antenna elements. When the array problem is considered to be a regularly spaced magnitude regulated situation with a uniform spacing ${d}_{u}$, the $AF$ of (3) will be minimized to

14 $$AF\left({I}_{n}\right)=2\sum_{n=1}^{N}{E}_{n}\mathrm{cos}\left\{\left(n-0.5\right)\beta {d}_{u}[\mathrm{cos}\left(\theta \right)-1]\right\}$$

Due to the symmetry of the magnitude of the array elements along the array axis, the overall number of feeding magnitudes to be optimized *(*${N}_{M}$*)* is only half the number of radiating components *(2N)*.

15 $${N}_{M}=({E}_{1},{E}_{2},{E}_{3},{E}_{4}, \dots ..{{E}_{N-1},E}_{N})$$

In this scenario, the LAA design is synthesized for magnitude regulation with fixed uniform spacing. As a result, selecting the most optimum value of uniform space is equally critical. The resulting radiation maxima for LAA point towards the broadsides of the array axis for a uniform separation of one wavelength in addition to the end fire direction. Furthermore, if the element spacing is half the wavelength, end-fire radiation arises on both ends ${(\theta }_{0}={0}^{\circ} \; \mathrm{ and } \; {\theta }_{0}={180}^{\circ}).$ Thus, to ensure the possibility of single end-fire maximum and no grating lobes, the spacing between antenna components should be smaller than half wavelength. As a result, uniform spacing of $0.25 \lambda$ is used in the EFA condition of this design to avoid any grating lobe. Furthermore, the controlled magnitudes are normalized to their greatest value to yield a range of 0 to 1.

**Figure 3:**
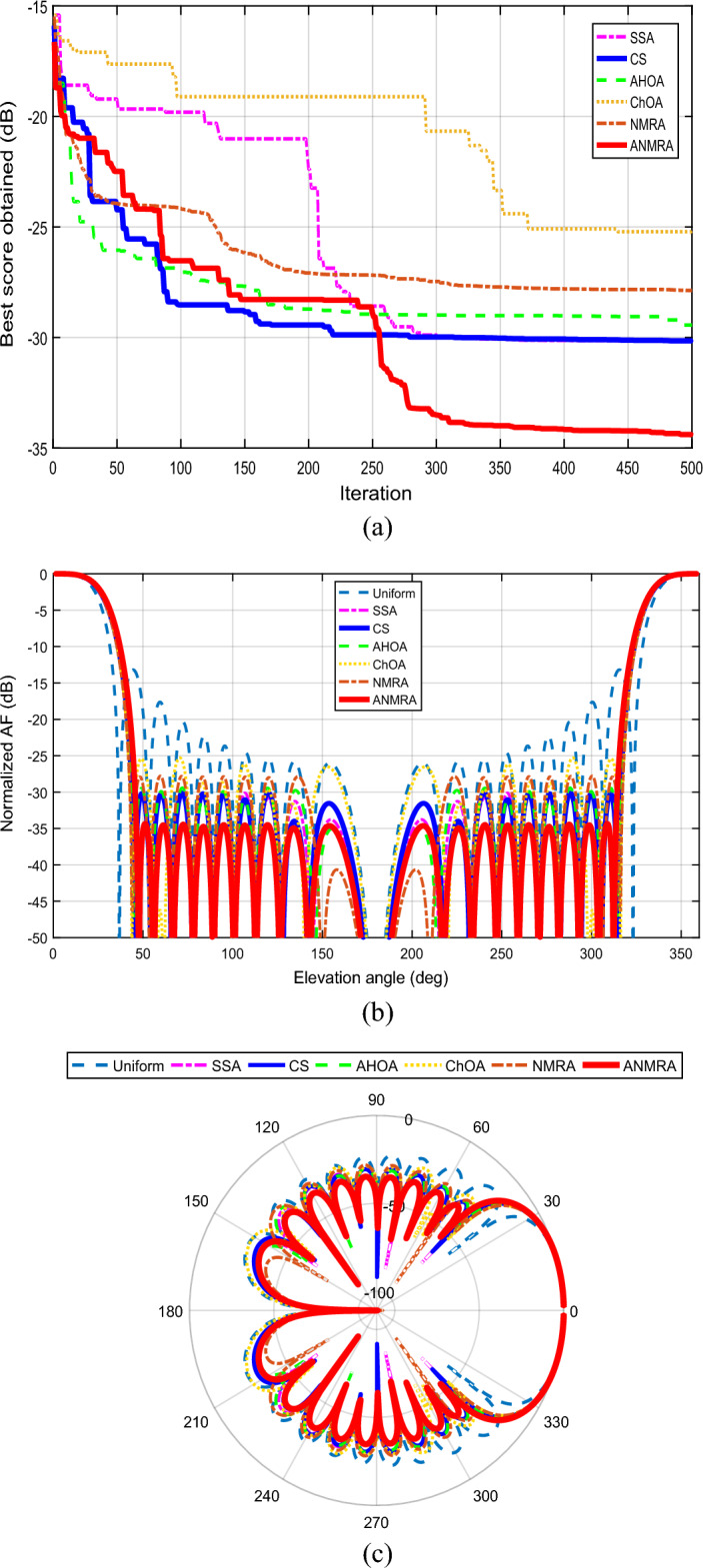
(**a**) Convergence graph for twenty elements magnitude control case. (**b**) 2D beam patterns for twenty elements magnitude control case. (**c**) Polar plot for twenty elements magnitude control case.

The convergence curve of the suggested fitness function for a 20 (N = 10) element EFA separated by equal intervals of $0.25 \lambda$ is shown in Fig. 3a. In relation to the other techniques, the ANMRA approach yields the best score for the specified objective function. The adaptive version of NMRA outperforms the basic algorithms. Figure 3b depicts the beam pattern obtained utilizing all of the mentioned methodologies. ANMRA has the lowest SLL, with a peak value of -34.43 dB, whereas SSA and CS have SLL levels that are identical at the completion of a maximum iteration. In comparison to the AHOA and NMRA, the ChOA has higher SLLs. Furthermore, as contrasted to the uniform magnitude case, all techniques performed brilliantly, although at the sacrifice of a wider primary lobe beamwidth. In the uniform magnitude case, the beamwidth is the narrowest, hence the highest directivity. As a result, there is a compromise between SLL and beamwidth in the magnitude control scenario. The primary lobe orientations and evaluation of grating lobe existence are equally important and are best shown on a polar graph, as seen in Fig. 3c. For all techniques, the radiation pattern's major lobe direction is parallel to the array axis (Z-axis) without any grating lobes. Tables 3 and 4 describe the controlled magnitude and main outcomes produced for this case.

**Table 3:** Controlled design parameters obtained using ANMRA for different LAA cases.

Controlling parameter	Location $[{z}_{1},{z}_{2},\dots . {z}_{N}]$ in $\lambda$	Normalized magnitude $\left[{{E}_{1},{E}_{2},\dots .E}_{n}\right]$
Magnitude only	[0.125,0.375,0.625,0.875,1.125,1.375,1.625,1.875,2.125,2.375]	[0.992,0.959,0.892,0.8,0.686,0.566,0.443,0.326,0.224,0.201]
Location only	[0.135,0.392,0.643,0.893,1.231,1.496,1.821,2.249,2.696,3.142]	[1,1,1,1,1,1,1,1,1,1]
Magnitude and location	[0.195,0.587,0.982,1.376,1.769,2.159,2.551,2.942,3.335,3.726]	[0.963,0.928,0.862,0.762,0.642,0.508,0.395,0.288,0.18,0.138]

**Table 4:** Key findings obtained for 20 element LAA for different controlling parameters.

Control	Magnitude only			Location only			Magnitude and location		
Algorithm	SLL (dB)	D (dB)	BW (deg)	SLL [dB]	D [dB]	BW [deg]	SLL [dB]	D [dB]	BW [deg]
Uniform	− 13.18	13.01	73.70	− 13.18	13.01	73.70	− 13.18	13.01	73.70
SSA	− 30.14	12.38	90.62	− 17.01	14.31	63.74	− 11.33	13.85	54.78
CS	− 30.15	12.37	90.60	− 17.79	13.42	71.70	− 12.10	12.97	84.66
AHOA	− 29.43	12.39	90.61	− 19.64	14.19	65.74	− 12.28	13.53	51.8
ChOA	− 25.34	12.46	87.64	− 18.80	13.97	68.72	− 23.31	13.98	71.7
NMRA	− 27.87	12.49	87.61	− 20.83	13.59	72.70	− 24.35	12.61	85.64
ANMRA	− 34.43	12.14	95.60	− 21.63	13.84	69.72	− 37.08	13.93	74.68

#### 2) LAA for location control only

This section analyses the EFA pattern of LAA employing just location control for feeding antenna components with homogeneous feeding magnitude. Assuming that the array structure is symmetric along the array axis, i.e. ${z}_{n}={z}_{-n}$ and stimulated by a normalized magnitude of unity, then the AF for determining controlled location may be described as follows:

16 $$AF\left({z}_{n}\right)=2\sum_{n=1}^{N}\mathrm{cos}\left\{\beta {z}_{n}[\mathrm{cos}\left(\theta \right)-1]\right\}$$

The total number of feeding component locations to optimize ${(N}_{L})$ is only half the number of antenna elements *(2N)* since the feeding elements are symmetric along the array axis.

17 $${N}_{L}=({z}_{1},{z}_{2},{z}_{3},{z}_{4}, \dots ..{{z}_{N-1},z}_{N})$$

The structure should be developed in such a manner that eliminates the likelihood of grating lobes. As stated earlier, the radiation pattern for EFA spaced by one wavelength provides peaks not only at the ends of the array axis but also on the broadside. Furthermore, if the element spacing is half the wavelength, end-fire radiation arises on both ends. As a result, to ensure that there is only one end-fire maximum and no grating lobes, the spacing between antenna elements should be smaller than half wavelength. Although, this is not the case in a non-uniformly spaced array. Thus, to widen the search span a maximum spacing of $0.95 \lambda$ is employed between neighboring antenna components. Similarly, $0.25 \lambda$ is set as the smallest spacing between array members. The design is now assessed using the required specifications and the same number of radiating components as considered in the previous magnitude control scenario.

**Figure 4:**
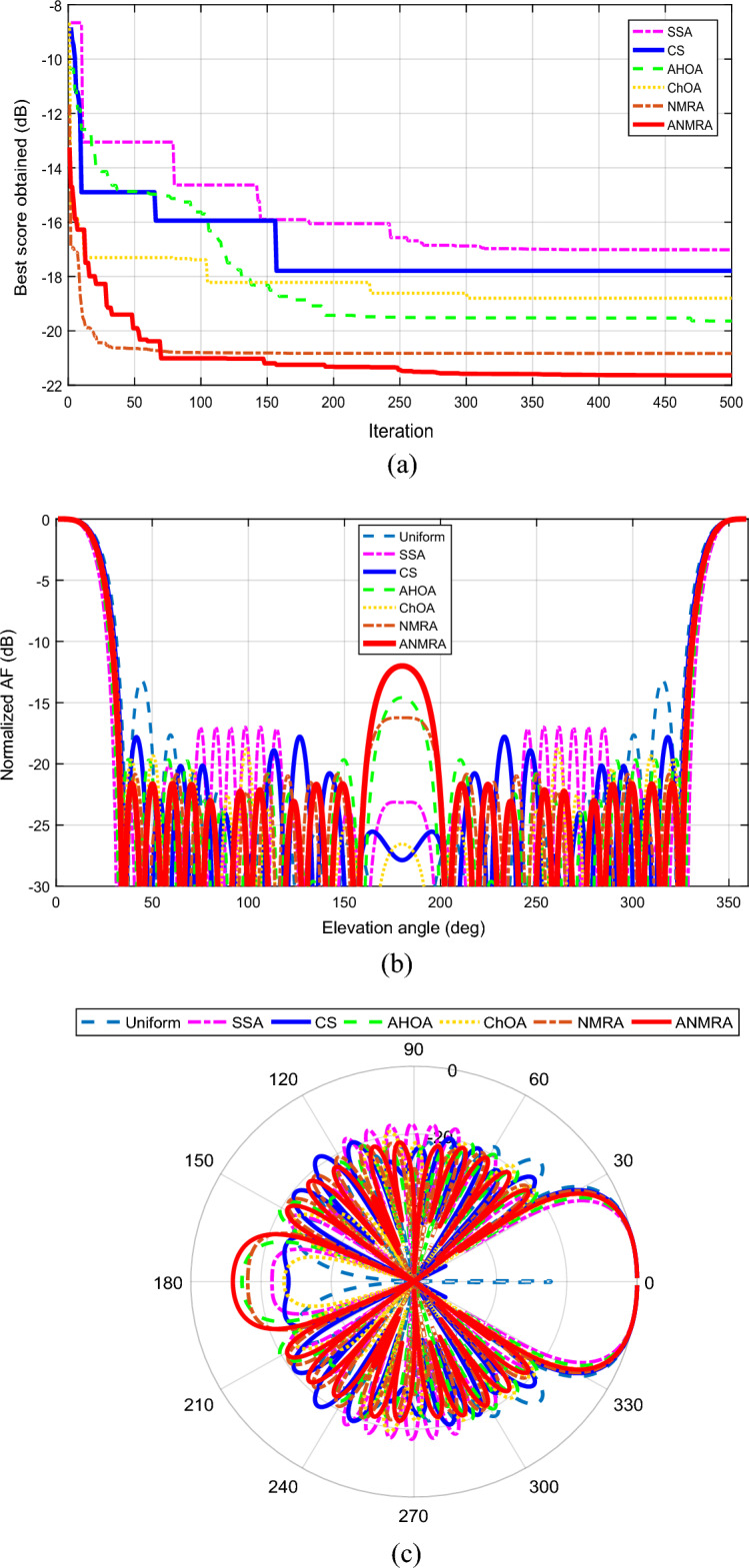
(**a**). Convergence graph for twenty elements location control case. (**b**) 2D beam patterns for twenty elements location control case. (**c**. Polar plot for twenty elements location control case.

Figure 4a depicts the convergence curve for the given scenario for a 20 element EFA driven by a homogeneous magnitude and optimum position. In comparison to the other techniques, the ANMRA produces the best score for the given situation. In this controlled scenario as well, the adaptive version of NMRA outperforms the original approaches. Figure 4b depicts the beam pattern obtained using all of the approaches. ANMRA has the least SLL, with a peak level of − 21.63 dB, whereas ChOA and AHOA have SLLs that are somewhat comparable with a peak value of − 18.80 and − 19.64 dB respectively. CS and SSA, on the other hand, have SLLs of − 17.79 dB and − 17.01 dB, respectively. In comparison to the uniform spaced case, all techniques performed better in terms of beamwidth. The beamwidth is greatest when the spacing is consistent. Although the shrinking obtained in beamwidth cost the SLL. Hence, in the location control scenario, there is a tradeoff between beamwidth and SLL. Figure 4c depicts the radiation pattern on a polar graph in addition to the SLL. The principal lobe direction of the radiation pattern is parallel to the array axis for all approaches. The polar graph reveals that the algorithms have secondary peaks at 180°. Even though subsidiary peaks are not equivalent to principal maxima, they do cause SLL deterioration near the lower end of the array axis. The controlled location and critical outcomes acquired for this instance are highlighted in Tables 2 and 3.

#### 3) LAA for magnitude and location control

This subsection investigates the LAA design for EFA radiation utilizing both magnitude and position control, with the goal of achieving optimum design parameters for minimal SLL and high directivity. The number of feeding magnitudes ($${N}_{M})$$ and locations *(*$${N}_{L}$$*)* to optimize is only half the number of antenna elements *(2N)* since the array elements' position and magnitudes are symmetric along the array axis. Thus, the total number of attributes to be optimized *(*$${N}_{i}$$*)* is the sum of $${N}_{M}$$ and $${N}_{L}$$, with the first half of the components representing excitation magnitude and the other half representing the element's location along the array axis.

18 $${N}_{i}=({E}_{1},{E}_{2},{E}_{3},{E}_{4}, \dots ..{{E}_{N-1},E}_{N},{z}_{1},{z}_{2},{z}_{3},{z}_{4}, \dots ..{{z}_{N-1},z}_{N})$$

Equation (3) is used to initiate the design, using the same magnitude and location conditions as used before with the identical number of radiating elements.

**Figure 5:**
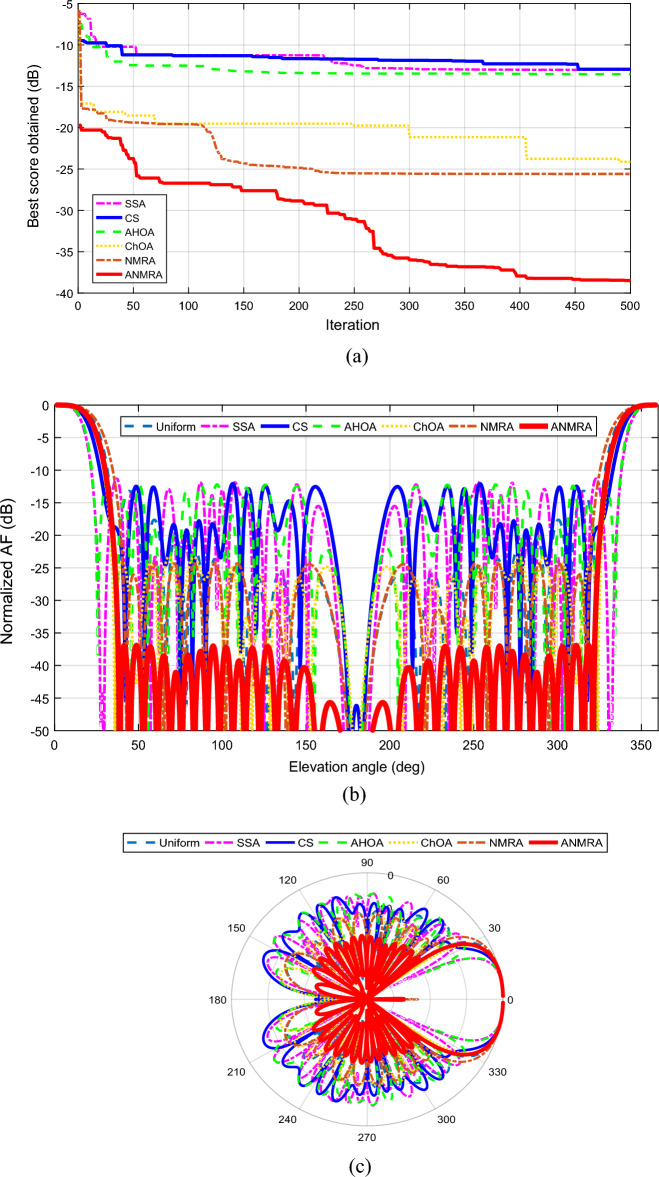
(**a**). Convergence graph for twenty elements magnitude-location case. (**b**) 2D beam patterns for twenty elements magnitude-location case. (**c**) Polar plot for twenty elements magnitude-location case.

The convergence graph for a 20 element EFA obtained by an optimized magnitude and optimized position in the present situation is shown in Fig. 5a. When compared to the other methodologies, the ANMRA delivers the lowest score in the current scenario. The adaptive version of NMRA clearly outperforms the original methods. The beam pattern achieved using all of the techniques is depicted in Fig. 5b. ANMRA has the lowest SLL, with a peak level of − 37.08 dB, whereas SSA, CS, and AHOA have SLLs that are almost comparable at the completion of maximum repetitions. ChOA and NMRA have SLLs of − 23.31 dB and − 24.35 dB, respectively. In addition to the SLL, a polar graph may be used to assess the existence of grating lobes, as illustrated in Fig. 5c. Furthermore, with regulated magnitude and location, the algorithm can diminish subsidiary maxima towards the lower end of the array axis without impacting SLL, as demonstrated in Fig. 5c. At 180° polar, the ANMRA algorithm's subsidiary maximum has been decreased to − 93.83 dB. Similarly, SSA, CS, AHOA, ChOA and NMRA have secondary maxima at 180° polar with maximum levels of − 87.21 dB, − 46.15 dB, − 56.51 dB, − 57.51 dB, and − 87.21 dB, respectively. The suggested ANMRA method has a narrow beamwidth equivalent to the uniform excitation situation, with an FNBW of 74.68°. As a result, this design situation can achieve the lowest SLL while maintaining beamwidth. Tables 2 and 3 illustrate the controlled location, magnitude, and key results acquired for this case.

#### 4) Comparative analysis for different controlling parameters of LAA

**Figure 6:**
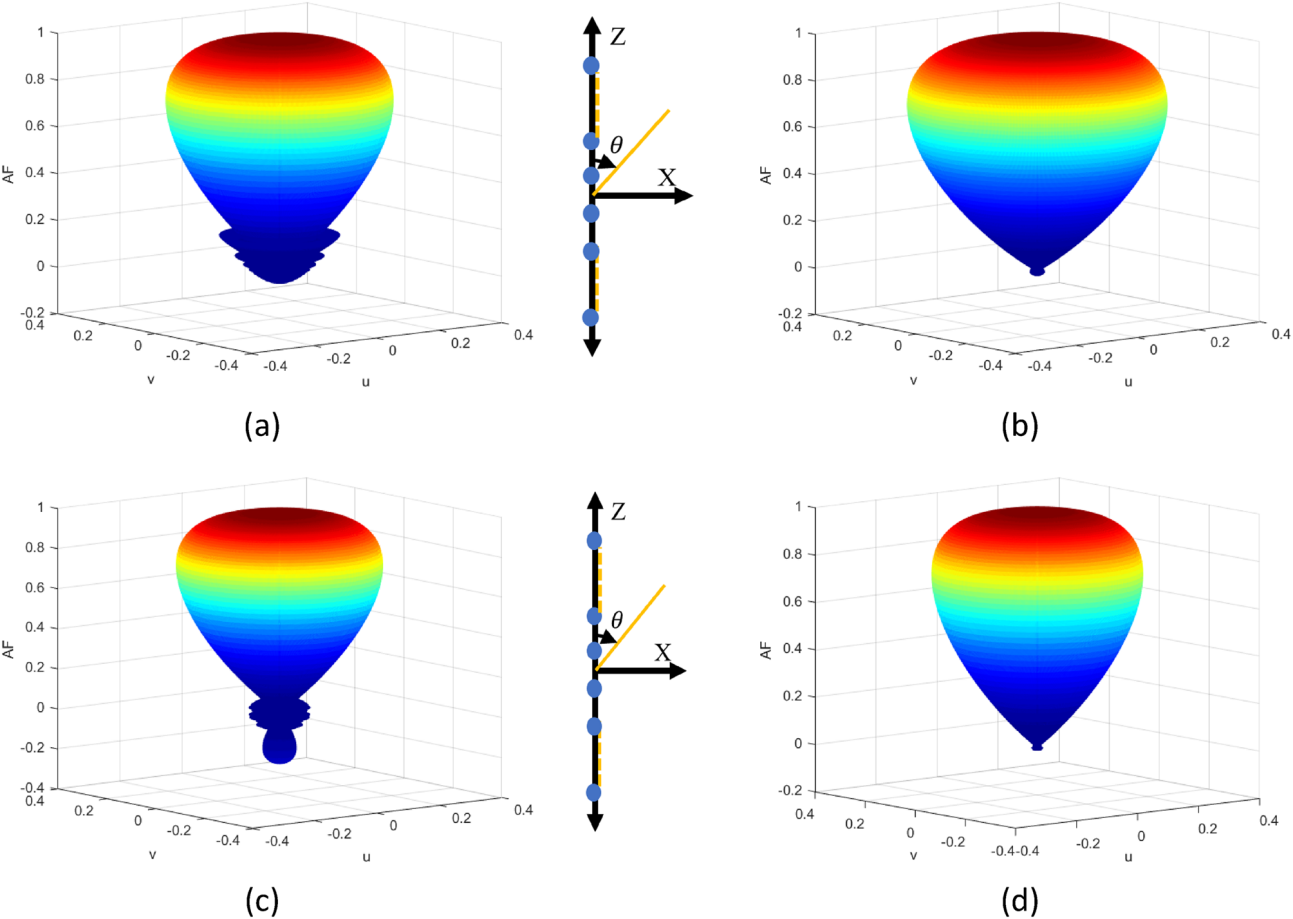
Twenty elements optimized LAA beam pattern in 3D using ANMRA for (**a**) uniform magnitude and location (**b**) non-uniform magnitude only (**c**) non-uniform location only (**d**) non-uniform magnitude and location.

This portion delves into the deep study of the various regulating parameters assessed in the preceding sections. Figure 6 depicts the 3D radiation pattern generated using ANMRA design synthesis for 20 components of LAA. The generated radiation for EFA has a high SLL for uniform magnitude and uniform spatial excitation, i.e. without optimum regulated parameters. However, when managing the LAA utilizing magnitude control exclusively with uniform space, minimal SLLs are seen. Figure 6b clearly shows that the beamwidth achieved for this instance is substantially thicker than the uniform excitation scenario for similar design parameters. Hence, a tradeoff is witnessed in which minimal SLL are obtained at the expense of beamwidth.

In contrast, the location only controls for the similar LAA design with uniform magnitude excitation shows a beamwidth somewhat smaller than the uniform excitation case. However, as seen in Fig. 6c, a few back lobes can be seen at the array axis' ends. As a result, beamwidth reduction comes at the expense of SLL. On a similar LAA design, the design is ultimately assessed for both magnitude and position control, and the radiation pattern obtained using the optimized magnitude and location is shown in Fig. 6d. This situation is seen to be able to break the beamwidth-SLL tradeoff. The beamwidth measured in this situation is substantially narrower than in the uniform excitation scenario, with essentially no SLL.

Table 4 summarizes the important numerical findings obtained for all three situations utilizing different metaheuristic algorithms for a better assessment of the proposed approach in comparison to other known techniques. It can be seen that for the magnitude regulated design of LAA, all of the applied approaches lower the SLL to substantial levels, with ANMRA achieving the lowest SLL of − 34.43 dB. However, the lower SLL is attained with a higher beamwidth, resulting in less directivity. The beamwidth achieved with a location controlled design is narrower than that produced with uniform stimulation. Nonetheless, the SLL obtained for the location control scenario is greater than the SLL obtained for the magnitude control scenario. In comparison to the magnitude control instance, ANMRA optimized regulated locations show a 12.8 dB deterioration. A few substantial level back lobes are also seen around the 180° elevation. Thus, a tradeoff between SLL and beamwidth is seen, with magnitude control achieving greater SLL and location control achieving narrower beamwidth. In this work, an attempt to simultaneously achieve low SLL with magnitude and smaller beamwidth with location is explored using these optimum conditions. To focus on obtaining low SLL while maintaining a small beamwidth, simultaneous magnitude and position optimization are used. In this scenario, a considerable drop in beamwidth due to location is achieved, combined with a substantial improvement in SLL due to magnitude. The ANMRA controlled design has the lowest SLL of − 37.08 dB and a small beamwidth of 74.68°. Furthermore, the proposed technology can totally eradicate the rear lobe. As a result, in comparison to the other technique, the suggested algorithm attains the lowest SLL while keeping a narrow beamwidth and high directivity. As a result, for all LAA criteria, the ANMRA algorithm reveals to be the best alternative.

## Conclusion

This study presents the use of the ANMRA algorithm for synthesizing LAAs with specific objectives such as principal lobe placement, grating lobe elimination, minimal SLL, small beamwidth and high directivity. The proposed ANMRA algorithm incorporates adaptive parametric settings and enhanced exploration and exploitation capabilities, resulting in improved performance and the ability to avoid local optima. The effectiveness of the proposed ANMRA algorithm is evaluated using the CEC 2019 benchmark test functions. To validate the statistical significance of the results, Wilcoxon rank-sum test and the Friedman rank (f-rank) test are conducted. The Wilcoxon rank-sum test is employed to compare the performance of the ANMRA algorithm with other methods, providing p-values that indicate the significance of the observed differences. The Friedman rank test is used to assign ranks to each algorithm based on its overall performance across the complete test suite. These statistical tests confirm the superior performance of the ANMRA algorithm. Through rigorous evaluations and comparisons with other established methodologies, including SSA, CS, AHOA, ChOA, and NMRA, this research assesses the design of antenna element magnitude and placement control. The objective function used in the analysis captures the tradeoff between SLL reduction and enhanced directivity, highlighting the need for careful optimization to achieve desired performance characteristics. When controlling only the magnitude of the LAA with uniform spacing, minimal SLL is achieved. However, this approach results in a wider beamwidth compared to the uniform excitation condition. On the other hand, adjusting the location of the elements alone yields a slightly lower beamwidth but introduces a few back lobes at the extremities of the array axis, affecting the SLL. The breakthrough comes when considering both magnitude and placement control simultaneously. This approach has the potential to break the beamwidth-SLL tradeoff, resulting in a narrower beamwidth, almost negligible SLL, and null at the lower end of the array axis. Compared to other methodologies, the proposed ANMRA algorithm demonstrates superior performance by minimizing SLL without the presence of grating lobes while maintaining a small beamwidth. In summary, the ANMRA algorithm offers a promising solution for managing the complex requirements of smart antenna system applications. It allows for efficient and effective synthesis of LAA designs, enabling enhanced beam directivity and communication performance. The findings of this research contribute to the advancement of smart systems communication technologies, facilitating the implementation of wireless networks in smart cities and other challenging environments.

## Data Availability

All data generated or analyzed during this study are included in this published article.
